# Stem Cell Derived Retinal Pigment Epithelium: The Role of Pigmentation as Maturation Marker and Gene Expression Profile Comparison with Human Endogenous Retinal Pigment Epithelium.

**DOI:** 10.1007/s12015-017-9754-0

**Published:** 2017-07-21

**Authors:** A. Bennis, J. G. Jacobs, L. A. E. Catsburg, J. B. ten Brink, C. Koster, R. O. Schlingemann, J. van Meurs, T. G. M. F. Gorgels, P. D. Moerland, V. M. Heine, A. A. Bergen

**Affiliations:** 1 Department of Clinical Genetics, AMC, Amsterdam, The Netherlands; 2 The Netherlands Institute for Neuroscience (NIN-KNAW), Royal Netherlands Academy of Arts and Sciences, Amsterdam, The Netherlands; 3 Department of Pediatrics/Child Neurology, VU University Medical Center, Amsterdam, The Netherlands; 4 Ocular Angiogenesis Group, AMC, Amsterdam, The Netherlands; 5 Department of Ophthalmology, AMC, Amsterdam, The Netherlands; 6 Department of Cell Biology and Histology, AMC, Amsterdam, The Netherlands; 7 Rotterdam Eye Hospital, Amsterdam, The Netherlands; 8 University Eye Clinic Maastricht, MUMC+, Amsterdam, The Netherlands; 9 Bioinformatics Laboratory, Department of Clinical Epidemiology, Biostatistics and Bioinformatics, AMC, Amsterdam, The Netherlands; 10 Department of Complex Trait Genetics, Center for Neurogenomics and Cognitive Research, Neuroscience Campus Amsterdam, VU University Amsterdam, Amsterdam, The Netherlands

**Keywords:** Human embryonic stem cells, Retinal pigment epithelium, Pigmentation, Age related macular degeneration, Cell replacement therapy, Transcriptomics

## Abstract

In age-related macular degeneration (AMD) the retinal pigment epithelium (RPE) deteriorates, leading to photoreceptor decay and severe vision loss. New therapeutic strategies aim at RPE replacement by transplantation of pluripotent stem cell (PSC)-derived RPE. Several protocols to generate RPE have been developed where appearance of pigmentation is commonly used as indicator of RPE differentiation and maturation. It is, however, unclear how different pigmentation stages reflect developmental stages and functionality of PSC-derived RPE cells. We generated human embryonic stem cell-derived RPE (hESC-RPE) cells and investigated their gene expression profiles at early pigmentation (EP) and late pigmentation (LP) stages. In addition, we compared the hESC-RPE samples with human endogenous RPE. We used a common reference design microarray (44 K). Our analysis showed that maturing hESC-RPE, upon acquiring pigmentation, expresses markers specific for human RPE. Interestingly, our analysis revealed that EP and LP hESC-RPE do not differ much in gene expression. Our data further showed that pigmented hESC-RPE has a significant lower expression than human endogenous RPE in the visual cycle and oxidative stress pathways. In contrast, we observed a significantly higher expression of pathways related to the process adhesion-to-polarity model that is typical of developing epithelial cells. We conclude that, *in vitro,* the first appearance of pigmentation hallmarks differentiated RPE. However, further increase in pigmentation does not result in much significant gene expression changes and does not add important RPE functionalities. Consequently, our results suggest that the time span for obtaining differentiated hESC-RPE cells, that are suitable for transplantation, may be greatly reduced.

**Electronic supplementary material:**

The online version of this article (doi:10.1007/s12015-017-9754-0) contains supplementary material, which is available to authorized users.

## Introduction

Regenerative medicine holds great promise for patients with degenerative diseases that are clinically characterized by tissue loss. Age-related macular degeneration (AMD) is a progressive degenerative disease and it is the leading cause of blindness in the elderly in the Western world. In people of 60 years of age or older, 4% is affected by a late severe stage of AMD [1]. AMD is classically characterized by the dysfunction and degeneration of the retinal pigment epithelium (RPE) in the macula, the part of the retina responsible for central vision. The RPE is a monolayer of cells in the back of the eye that plays an important role in the maintenance and health of the photoreceptors [2, 3].

AMD presents itself in two forms: wet and dry. The more severe wet form accounts for 10–15% of the cases [4], and is characterized by neovascularization. This form can be treated by monthly intra-ocular injections of anti-angiogenic drugs. Even though frequently effective, this is a patient unfriendly, invasive and costly treatment. Dry AMD is more prevalent and is characterized by a slow buildup of yellowish deposits beneath the RPE, called drusen, which progresses to geographic loss of RPE and subsequently photoreceptor atrophy. There are several treatment options for dry AMD, including RPE transplantation, laser photocoagulation, photodynamic therapy, submacular surgery, transpupillary thermotherapy, and pharmacotherapy [5–8]. However, these approaches are not very effective, and thus there is much interest in the development of new therapies.

AMD is a genetically complex disorder, and, at least in the classical view, the primary pathology is limited to a single cell type (the RPE). RPE transplantation may be the only AMD treatment that can restore the function of already degenerated cells, if this is performed in an early stage of AMD in order to prevent photoreceptor loss. However, replacement of degenerated tissue with donor material, or the translocation of autologous RPE sheets from the periphery to the macula, have had limited success so far [6, 9]. This can partly be ascribed to the technical challenges involving the collection of sufficient tissue, transplant rejection, and the difficulties in controlling harvest and direct use of age- and genetically-matched cells.

The use of pluripotent stem cell derived-RPE cells (PSC-RPE) may circumvent some of these problems, as we have more and more control of generating specific neural subtypes, such as RPE, using HLA-matched PSC sources and scaling cell products to sufficiently high numbers.

Several groups recently optimized PSC differentiation protocols to generate RPE. Early protocols were based on so-called spontaneous differentiation by letting PSC freely differentiate using the adherent culture or floating embryoid body methods into pigmented RPE cells [10–12]. Although these protocols reliably produce pigmented cells, they are time-consuming and inefficient. Later protocols, so called the directed differentiation methods, showed improved efficiency. Directed differentiation methods use the addition of growth factors to induce RPE differentiation, and either involve adherent, suspension or 3D cultures to resemble the *in vivo* development more closely (reviewed by Leach et al. 2016 [13]).

Although we are able to generate RPE(−like) cells *in vitro*, our knowledge about the most suitable differentiation state and corresponding function before and upon transplantation is limited. So far the emergence and increase of pigmentation is used as important hallmark for differentiation and further maturation of PSC-RPE. It is however unclear how the PSC-RPE changes during this increase in pigmentation, how PSC-RPE with little pigmentation compares to PSC-RPE with much pigmentation, and to what extent they represent stages in maturation towards the human endogenous RPE.

We adapted an established directed differentiation protocol to produce human embryonic stem cells derived-RPE cells (hESC-RPE) [14]. Subsequently, we compared the gene expression profiles of hESC-RPE samples that start to show pigmentation and that of samples that are almost fully pigmented. Finally, we compared the hESC-RPE samples to endogenous human RPE.

## Materials and Methods

### Maintenance of hESC Cells and RPE Differentiation

hESC line H1 (WA01, WiCell Research Institute, Madison, USA) was cultured in Essential 8 medium (Thermo Fischer, Waltham, USA) on Geltrex LDEV-Free hESC-qualified Reduced Growth Factor Basement Membrane Matrix (Thermo Fischer, Waltham, USA) coated 6-well plates. The cells were passaged as clumps every 3 to 4 days using 0.5 mM UltraPure EDTA (Thermo Fischer, Waltham, USA) dissolved in DPBS without Calcium and Magnesium (Thermo Fischer, Waltham, USA). Morphologically distinguishable differentiated cells were mechanically removed at each passage. To improve cell survival during passaging, the Rho kinase inhibitor, Y-27632 (SelleckChem, Houston, USA), was added in the culture medium during the first 24 h after plating.

To produce hESC-RPE cells, undifferentiated cell colonies were partially lifted by EDTA and scraped off with a cell scraper. The cell aggregates (150–250 um diameter) from one well of a six-well plate that was densely packed with colonies, were embedded in 150-250ul Matrigel (Corning, Corning, USA). The Matrigel containing the cells was plated 150ul per well on a six wells plate. They were plated as drops of Matrigel without touching the sidewalls of the wells. After gelling at 37 °C for 10 min, neural induction medium N2B27 was added, prepared as described (Pollard, Benchoua and Lowell 2006 [15]). After three days of differentiation, the cells were taken out of the Matrigel using Cell Recovery Solution (Corning, Corning, USA). To make single cells from the three-dimensional spheroids we treated it with TrypLE Express (Thermo Fischer, Waltham, USA), followed by gentle trituration. The cells were resuspended in N2B27 medium, containing 10 uM Rho kinase inhibitor to promote cell survival and seeded onto growth factor reduced Matrigel (Corning, Corning, USA) coated 6.5 mm Transwell inserts with 0.4uM pore polyester membrane (Corning, Corning, USA), at a density of 2-4 × 10^5^ cells/insert. At day 4 the cells were washed with RPE medium (see Zhu et al. 2013 for details [14]) and were kept in culture with RPE medium that contained human Activin A (100 ng/ml) (Agrenvec, Madrid, Spain). RPE medium consists of DMEM/F-12; no glutamine supplemented with 20% KnockOut Serum Replacement; MEM Non-Essential Amino Acids Solution; GlutaMAX Supplement; 100 U/ml Penicillin-Streptomycin and 0.1 mM 2-Mercaptoethanol (All from Thermo Fischer, Waltham, USA). Medium was changed every 2–3 days.

### RNA Isolation and (sq)RT-PCR

Total RNA was isolated using the RNeasy Micro Kit (Qiagen, Hilden, Germany). Subsequent reverse transcription to cDNA was performed with Superscript III reverse transcriptase (Life Technologies, Waltham, USA). The synthesized cDNA was amplified with transcript specific, intron-spanning primers (See Table S1 for the primer sequences). PCR was carried out with HOT FIREPol DNA Polymerase (Solis Biodyne, Tartu, Estonia) with an annealing temperature of 60 °C and 33 cycles. For the sqRT-PCR’s, we calculated the relative abundance of transcript expression by quantifying the gene expression in ImageJ and normalizing it to the housekeeping gene β-actin (*ACTB).*

### Immunocytochemistry

Cells were fixed with 2% paraformaldehyde for 20 min at room temperature, followed by blocking with 0.1% BSA, 0.3% Triton X-100, 5% normal goat serum, in 1× PBS. Incubation with the primary antibodies was performed in blocking buffer and done overnight at 4 °C. The working solutions were as follows: rabbit anti-RLBP1 1:200 (PA5–29759, Thermo Fisher, Waltham, USA), rabbit anti-MITF 1:200 (PA5–38294, Thermo Fisher, Waltham, USA), rabbit anti-ZO1 1:100 (61–7300, Thermo Fisher, Waltham, USA), rabbit anti-BEST1 1:100 (ab14928, Abcam, Cambridge, UK). The immunoreactivity of the antibodies was confirmed by immunostainings on human retinal cryosections and ARPE19 cells as positive control (Fig. S1). As a secondary antibody we used the Alexa Fluor 594 goat-anti-rabbit 1:1000 (A-111012, Thermo Fisher, Waltham, USA). Cell nuclei were counterstained with DAPI (Thermo Fisher, Waltham, USA). Cells were imaged using a Leica TCS SP8 X confocal microscope.

### Microarray Sample Collection and Preparation

We selected two microarray sample groups based on their pigmentation state during the hESC-RPE differentiation protocol. For six independent differentiation experiments we harvested cells, when the cells in the inserts started to show pigmentation (timepoint “Early Pigmentation”, EP) and when they were more than 80% pigmented (timepoint “Late Pigmentation”, LP), measured in ImageJ. The average days in culture for the EP samples is 32 (*s* = 8.6), and for the LP samples 62.5 (*s* = 12.1). We used global (manual) thresholding to determine the percentage of pigmented area. Photographs of the inserts were made with an 8-megapixel phone camera. These were loaded into ImageJ and converted to 8-bit images in order to be able to segment the image. The membrane of the insert was selected to include the whole culture surface. By thresholding the area that contains pigmented cells was included in the percentage. Because of variation in lighting of the original photos, we determined the threshold independently for every sample.

RNA isolation, amplification and labelling procedures were carried out essentially as described elsewhere [16]*.* Quality of the total RNA was checked with a Bioanalyzer assay (RNA 6000 Pico Kit, Agilent Technologies, Amstelveen, The Netherlands). The average RIN value for the total RNA of both the EP and the LP samples was 9.7, indicating excellent quality. In our microarray study we used a common reference design. As a common reference we used RNA from human RPE/choroid that was used in previous and on-going gene expression analyses in our lab [16, 17]. In short, the common reference sample consists of RNA from a pool of RPE/choroid isolated from 10 donor eyes (mean age 60 years). It was prepared using the same methodology as our experimental samples, and labelled with Cy3 (Cy3 mono-reactive dye pack, GE Healthcare UK, Little Chalfont, Buckinghamshire, UK). See Janssen et al. (2012) [16] for a more detailed description RNA processing and microarray procedures.

In addition, to make sure we compared hESC-RPE cells, we performed a RT-PCR experiment (Fig. S2). We studied the expression of *RAX, VSX2, MITF, TYR, TRPM3, TJP1, RLBP1, RPE65, MERTK* in EP and LP samples. The results confirmed the RPE character of the cells.

### Microarray Data Analysis

The microarray data were extracted using Agilent Feature Extraction Software (Agilent Technologies, version 9.5.3.1). Raw data were imported into R (version 2.14.0 for Windows, R Development Core Team, 2009) using the Bioconductor package LIMMA. Background correction was performed using the “normexp” method with an offset of 10 to adjust the foreground signal without introducing negative values. The resulting log-ratios were transformed using intensity-dependent loess normalization. We further normalized the average intensities across arrays using the Aquantile method [18].

The microarray data is available in the Gene Expression Omnibus database with the accession number GSE85907.

Genes that are differentially expressed between the EP and LP hESC-RPE, or between the hESC-RPE (EP and LP) and human endogenous RPE, were identified on the normalized log-ratios using a linear model. The data for the human endogenous RPE were derived from a previous study that used the exact same microarray strategy and analysis (submitted). This dataset consists of 5 independent donor eyes that were enucleated and snap-frozen within 24 h post mortem. The eyes were stored at −80 °C until use. Donors were aged 49 to 73 at time of death. Donors were selected for not having any ophthalmic disorder and visual inspection examination showed no retinal pathology. To collect the RPE, a macular fragment of 16mm^2^ with the fovea in its center was cut from the retina.12uM Sections from the macular area were used to isolate the RPE cells [19]. The sections were dehydrated with ethanol and air-dried before micro dissection. To minimize cellular cross-contamination in our procedure, we used the meticulous laser dissection microscope to cut the RPE monolayer specifically (PALM Carl Zeiss, MicroImaging GmbH, Munich, Germany).

Significant differences were determined using Bayes moderated paired t-statistics (package LIMMA in R). Resulting *p*-values were corrected for multiple testing using Benjamini-Hochberg False Discovery Rate adjustment. To identify specific differences between the EP hESC-RPE and the LP hESC-RPE, we used cutoff values of a fold change (FC) >2.5 and a *p*-value < 0.05. We found 246 genes significantly higher expressed in the EP hESC-RPE and 65 genes significantly higher expressed in the LP hESC-RPE.

Subsequently, we statistically tested the differences between the hESC-RPE (EP and LP) and human endogenous RPE. We used the stringent cut off values of FC > 5 and adjusted *p* value of *p* < 0.001 because we were interested in the most significantly specific differences between the two groups. This resulted in 737 genes significantly higher expressed in the hESC-RPE (EP and LP) and 1022 genes significantly higher expressed in the human endogenous RPE.

To investigate the degree of equality between gene expression profiles of the various groups, we plotted the samples on a multidimensional scaling plot (two dimensions) in the LIMMA package in R. The purpose of this plot is to provide a visual representation of the pattern of proximities (i.e. similarities or distances) among a set of objects. Those objects that are perceived to be very similar to each other are placed near each other on the map, and the objects that are perceived as very different are placed far away from each other.

Functional annotation was done in IPA, Ingenuity (Ingenuity Systems, version 24,718,999, assessed at May 31st, 2016). To present the results as comprehensive as possible we highlighted only the Ingenuity canonical pathways because these depict the most simple and straightforward representation of our data and functionalities.

### Confirmation of Microarray Results

We confirmed our microarray data with sqRT-PCR (Fig. S3). sqRT-PCR was carried out using intron-spanning primers on cDNA from EP and LP, using 6 biological replicates. To minimize effects of RNA degradation artefacts, we generated primers near the 3’end of the gene. We quantified the gene expression in ImageJ.

## Results

### Characterization of hESC-RPE Differentiation

**Fig. 1:**
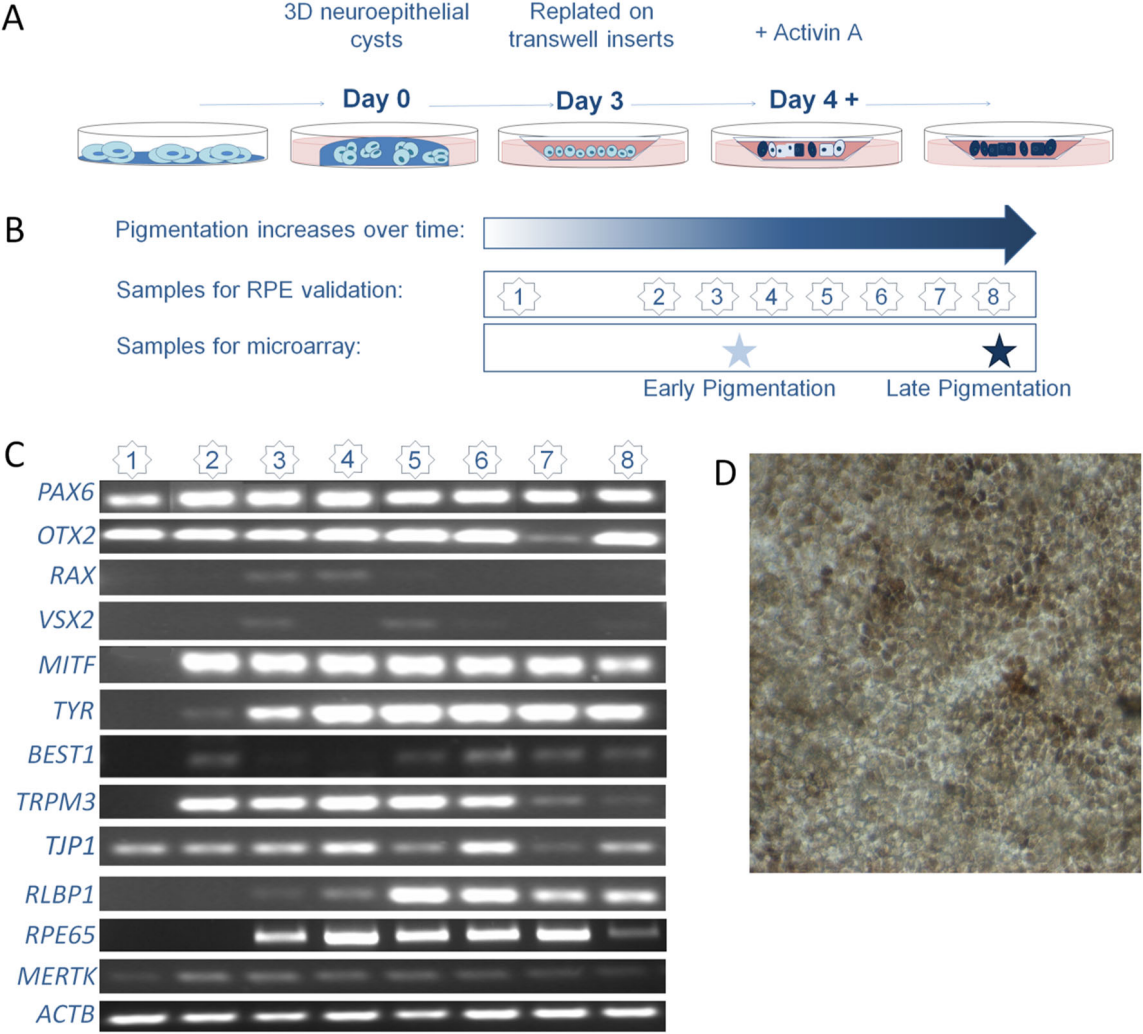
(**a**) Overview of the hESC-RPE differentiation protocol adapted from Zhu et al. [14]. (**b**) Scheme shows the different time points for collection of samples for validation of hESC-RPE generation (1 = 3 days, 2 = 10–12 days, 3 = 20–25 days, 4 = 30–35 days, 5 = 40–45 days, 6 = 50–55 days, 7 = 60–63 days, 8 = 70 days), by RT-PCR analysis. We also collected RNA when the cells started to show pigmentation (EP) and when more than 80% of the confluent culture was pigmented (LP). (**c**) RT-PCR analysis at time points 1–8 showed absence and expression of characteristic RPE genes. (**d**) The hESC-RPE cells started to show first pigmentation phenotypes and typical epithelial hexagonal morphology at timepoint 4

**Fig. 2:**
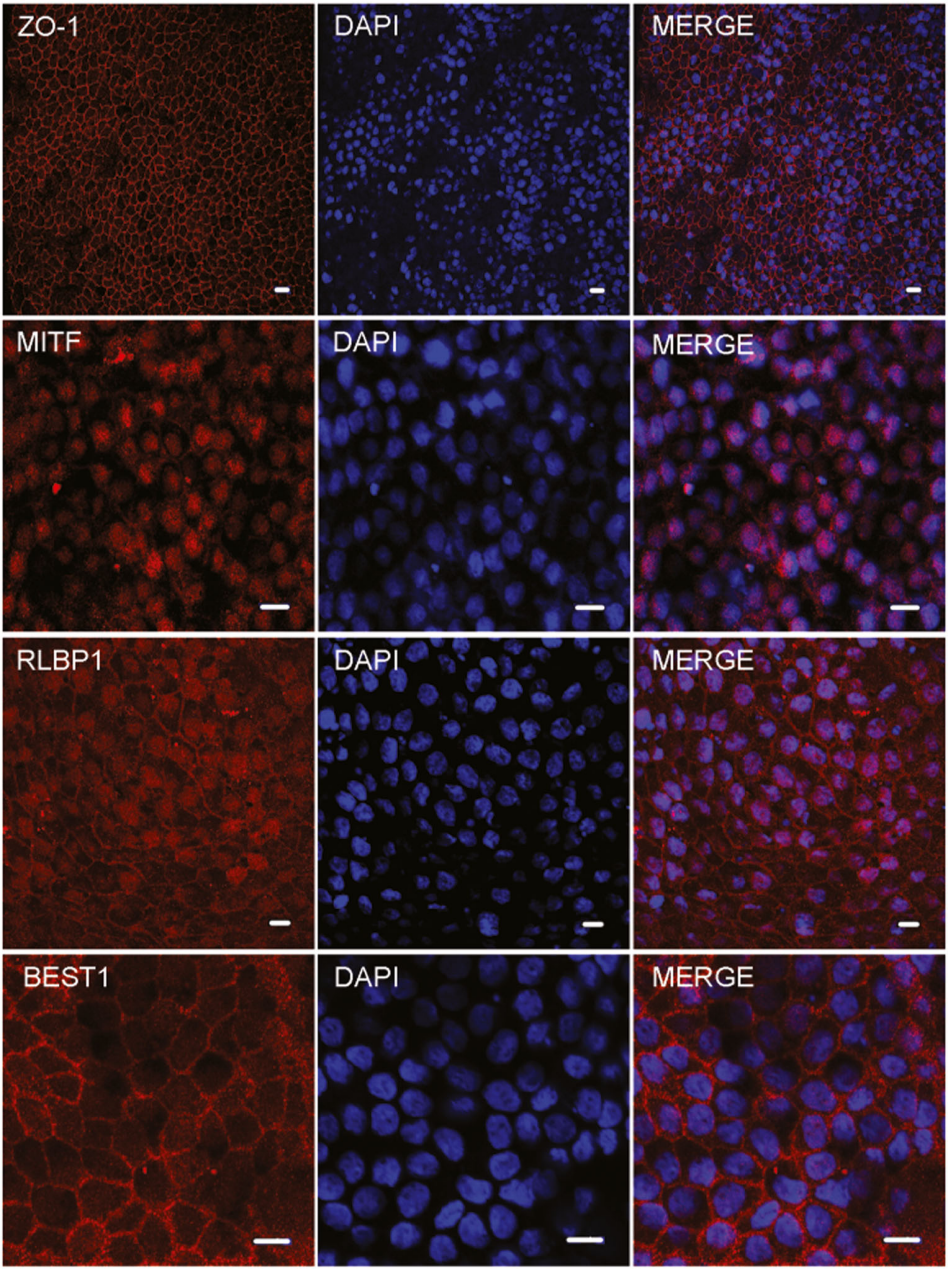
RPE generation was confirmed by immunocytochemistry for the tight junction protein ZO-1, transcription factor MITF, visual cycle related protein RLBP1 and the chloride channel BEST1 (scalebar = 10uM)

We differentiated hESC into RPE cells according to an adapted protocol previously described by Zhu et al. 2013 [14] (Figure 1A). We reduced the incubation time of the three-dimensional spheroids in the Matrigel from 5 to 3 days as in our hands the spheroids were already fully grown within 3 days. To confirm RPE development, we performed RT-PCR at different time points during hESC-RPE cell generation (Figure 1B). We measured gene expression of well-known RPE markers in our hESC-RPE cells at several time points (Figure 1C) [2]. Before pigmentation (time point 1 and 2), hESC-RPE expressed the early eye development markers *PAX6* and *OTX2*, which stay present till late differentiation stages (Figure 1C). By early onset of pigmentation (time point 2 and 3), most RPE-specific genes are turned on (*MITF, TYR, BEST1, TRPM3, RLBP1, MERTK, RPE65 and TJP1*). In our differentiation protocol, the early eye marker *RAX* is only clearly expressed at time points 3 and 4, but that does not seem to hinder the expression of other important RPE developmental genes (see previous sentence). RT-PCR analysis also confirmed the generation of the RPE by almost complete absence of *VSX2*, a marker for retinal progenitor cells. We see some VSX2 expression at time points 3 and 5, that disappears at later stages. This transient expression level of VSX2 may indicate the switching point between the development of photoreceptors or RPE [20]. In addition, as many PSC-derived protocols are challenged by high variability, we measured 50 independent samples, derived from 16 independent differentiation procedures, for a (semi-) quantification of the data after normalization of the expression to the housekeeping gene *ACTB* (Fig. S4). We found a high amount of variation. Generation of RPE-like cells was further shown by light microscopy analysis, identifying typical epithelial cobblestone RPE-like appearance and the presence of pigment granules (Figure 1D), and by immunocytochemical analysis of RPE-specific markers ZO-1, MITF, RLBP1 and BEST1 (Figure 2). Additionally, hESC-RPE showed photoreceptor outer segment phagocytosis using a previously published protocol (Fig. S5) [21, 22].

### Gene Expression Profile Analysis of Early and Late-Stage Pigmentation of hESC-RPE

To investigate RPE maturity and functional properties of EP and LP hESC-RPE in more depth, we performed six independent experiments (see Materials and Methods for details). These samples were used for a microarray study.

After feature extraction, we performed a paired t test on the gene expression data of the two groups (EP and LP hESC-RPE) and made a selection using a Benjamini-Hochberg (B-H) corrected *p* value <0.05 and fold change >2.5. We found a total of 311 genes differentially expressed (Table S2). Even though the sample groups were determined by their pigmentation levels, there are no genes in this list that are well-known for the melanogenesis in the RPE [23, 24]. The expression levels of these melanogenesis genes (*PAX6, OTX2, TYR, TYRP1, DCT, MITF, SI, MLANA*) are comparable between EP and LP samples (for details see the normalized expression levels of the microarray at the Gene Expression Omnibus database, accession number GSE85907).

**Fig. 3:**
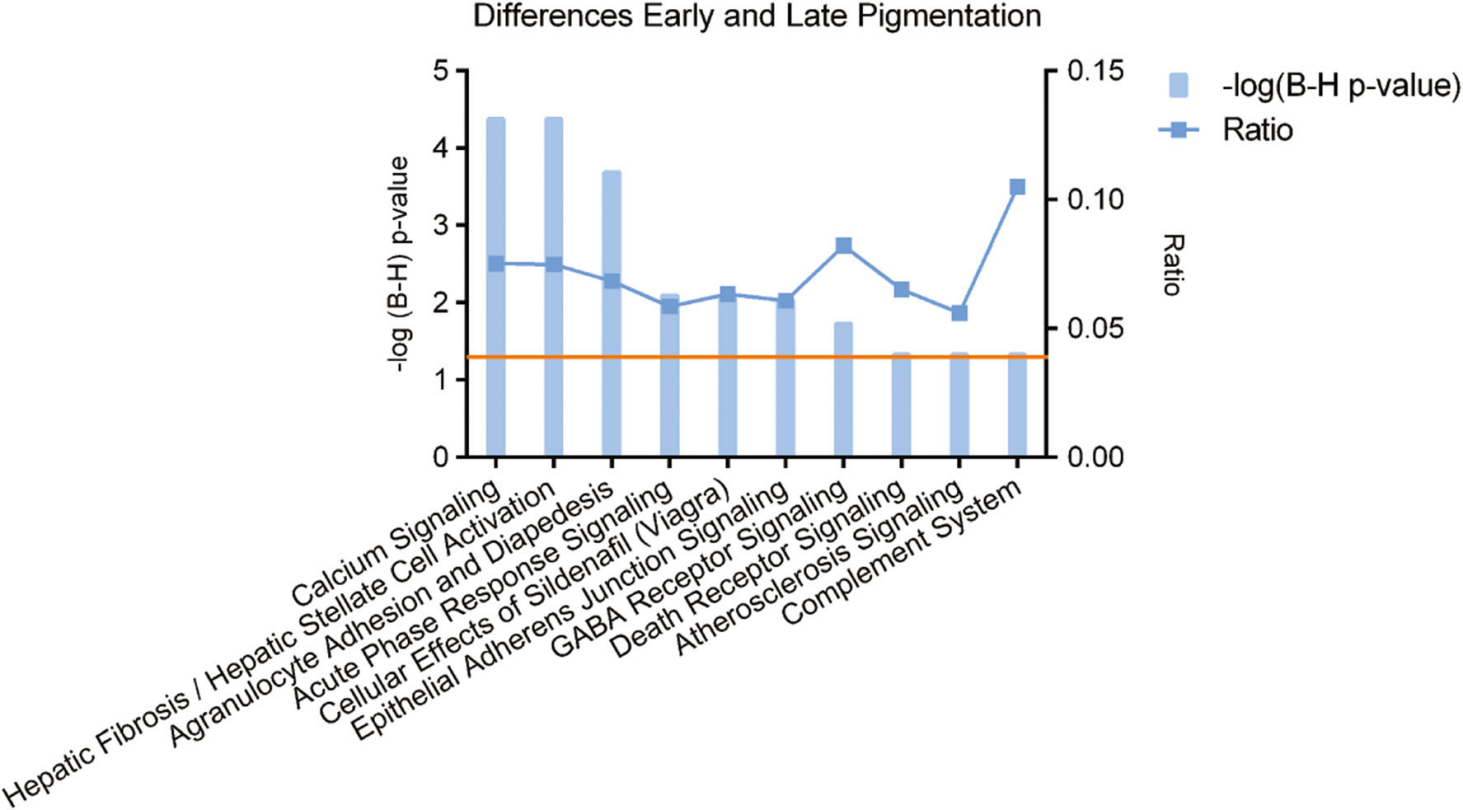
Canonical pathways identified by IPA for the genes that are significantly differentially expressed genes between EP and LP samples. The left y-axis displays the –log of the Benjamini-Hochberg corrected –value. The right axis displays the ratio of the number of genes derived from our dataset, divided by the total number of genes in the pathway. The bar graph represents the –log(B-H) *p*-value. The orange line indicates the threshold at a B-H corrected *p*-value <0.05

Subsequently, we used the IPA knowledge database to attribute a selection of overrepresented pathways to the differences between EP and LP hESC-RPE cells. These functions are depicted in Fig. 3.

**Fig. 4:**
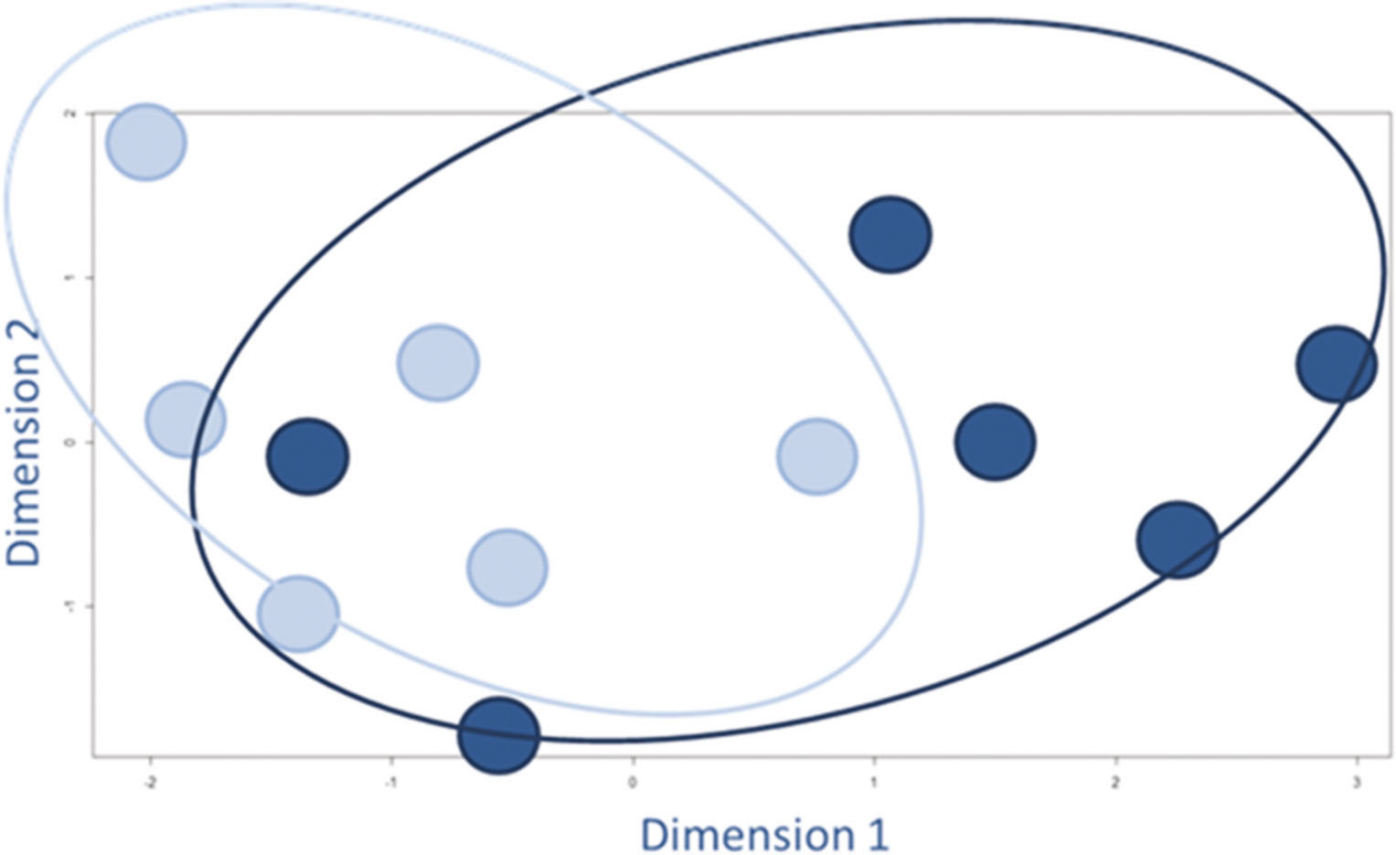
Multidimensional scaling plot to visually represent the (dis)similarities among the different hESC-RPE cell samples. The light blue dots represent the individual EP samples and the dark blue dots represent the LP samples. We used the LIMMA package in R, which is specific for the analysis of microarray data, and included all the normalized expression data of the individual samples: 43,376 entries per sample

Because only a relatively small number of genes showed statistically significant differences (311 out of 19,596 unique genes on array), we also analyzed the (dis)similarities of the *overall* expression of the individual samples. We plotted the normalized expression data (this includes the expression of all the entries that are measured on the array: 43,376 entries per sample) in a multidimensional scaling plot to visualize the level of (dis)similarity (Figure 4). This plot showed no clear segregation between the EP and LP hESC-RPE groups.

### Comparison of hESC-RPE and Human Endogenous RPE Expression Profiles

**Fig. 5:**
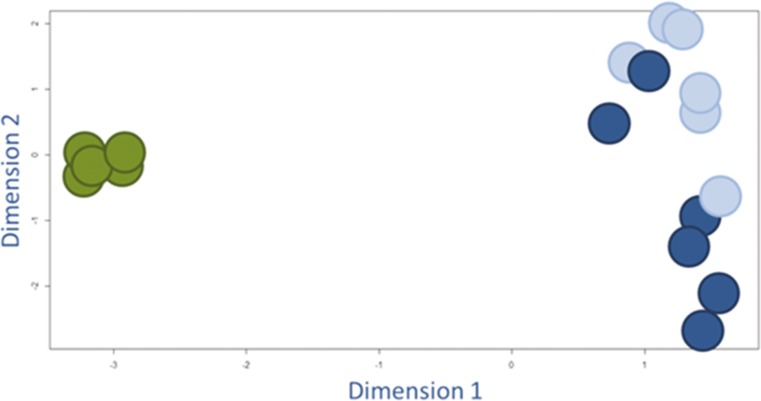
Multidimensional scaling plot to visually represent the (dis)similarities among the different hESC-RPE cells (blue dots) and human endogenous RPE (green dots). Also see Fig. 3

Next, we studied how similar the *in vitro* cultured hESC-RPE cells are to human endogenous RPE. EP and LP hESC-RPE did not show clear differences and we combined the data into one hESC-RPE group. We compared that group with human endogenous RPE gene expression data, previously generated from laser-dissected RPE from human donor eyes, using the same microarray platform and common reference design (submitted). To begin, we analyzed the (dis)similarities of the *overall* expression of the individual sample using multidimensional scaling (Figure 5).

The multidimensional scaling plot shows that the overall expression profiles are very different between hESC-RPE and the human endogenous RPE sample groups. This analysis also shows that there is more variation within the hESC-RPE sample group than within the human endogenous RPE samples group.

**Fig. 6:**
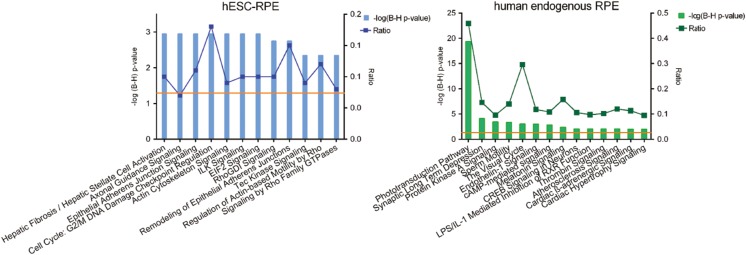
Canonical pathways identified by IPA for the genes that are significantly differentially expressed between the hESC-RPE cells and the human endogenous RPE. The left graph (blue) depicts the canonical pathways that relate to the genes specifically expressed in the hESC-RPE (this study). The right graph (green) depicts the canonical pathways that relate to the genes specifically expressed in the human endogenous RPE (submitted). In the graphs, the left y-axis displays the –log of the Benjamini-Hochberg corrected –value. The right axis displays the ratio of the number of genes derived from our dataset, divided by the total number of genes in the pathway. The bars show the –log(B-H) *p*-value. The orange line indicates the threshold at a B-H corrected *p*-value <0.05

To further compare the hESC-RPE and human endogenous RPE, we performed an unpaired t test. Here we considered genes significantly differentially expressed with a B-H adjusted *p* value <0.001 and fold change >5. We chose these stringent cutoff values in order to focus on the most prominent differences. We found 737 genes significantly higher expressed in the hESC-RPE (EP and LP) cells compared to the human endogenous RPE and 1022 genes significantly higher expressed in the human endogenous RPE compared to the hESC-RPE (Table S3). We conducted a functional annotation in IPA for the differentially expressed genes between the hESC-RPE (EP and LP) samples and the human endogenous RPE (Figure 6). This yielded 12 canonical pathways higher expressed in the hESC-RPE (EP and LP) cells, of which eight pathways are related to the so called adhesion-to-polarity model: Epithelial Adherens Junction Signaling, Actin Cytoskeleton Signaling, ILK Signaling, RhoGDI Signaling, Remodeling of Epithelial Adherens Junctions, Tec Kinase Signaling, Regulation of Actin-Based Motility by Rho, Signaling by Rho Family GTPases. The analysis in IPA resulted in 14 canonical pathways that are higher expressed in the human endogenous RPE. Most prominent was the appearance of pathways related to the visual system: Phototransduction Pathway and The Visual Cycle. Other pathways were relevant to oxidative stress handling: Protein Kinase A Signaling, cAMP-mediated Signaling, CREB Signaling in Neurons, Melatonin Signaling. And also maintenance of the blood-retina-barrier: Endothelin-1 Signaling and Thrombin Signaling.

## Discussion

In this study we expanded our knowledge on the development of hESC-RPE cells and generated expression profiles of EP and LP hESC-RPE samples, to investigate the suitability of pigmentation as a maturation marker in hESC-RPE differentiation. In addition, we compared the gene expression profiles of the hESC-RPE cells and the human endogenous RPE that it is supposed to replace.

We generated functional hESC-RPE cells using a well-established directed differentiation protocol. As many human stem cell-derived cultures are challenged by high amounts of variation, hESC-RPE cultures do not always mature with the same speed. Consequently, virtually all RPE differentiation studies use pigmentation as a maturation marker for the culture instead of time. This seems like a reliable benchmark and easy to use because it is clearly visible.

In attempt to answer the question whether increasing pigmentation indicates differentiation into more mature hESC-RPE cells, we performed a microarray study with the EP and LP hESC-RPE samples. In the comparison we found only a small amount of statistically significant differences. This implies that EP and LP hESC-RPE samples may be less different than generally accepted. Even though pigmentation seems to be a good biomarker for RPE development, the level of pigmentation does not reflect the maturation state of hESC-RPE. In terms of gene expression profile and functional annotation, cells seem to be at a similar developmental stage at EP and LP. Both the EP and LP cells show the expression of well-known RPE markers which is an important prerequisite for the transplantation of PSC-RPE cells [14, 25–30]. This could mean that there is no need to wait for the cells to be fully pigmented because it does not make a substantial difference.

To be able to say more about how the hESC-RPE cells compare to human endogenous RPE, we subsequently compared the gene expression profiles of the hESC-RPE (EP and LP) samples and the gene expression profiles of human endogenous RPE samples.

In our analysis we found 12 canonical pathways highly expressed in the hESC-RPE (EP and LP) as compared to the human endogenous RPE. It is striking that eight of these are involved in the adhesion-to-polarity model that is typical for developing epithelial cells. The human endogenous RPE is a highly polarized cell type with distinct apical and basolateral plasma membrane domains. Cell polarity is initiated through a combination of spatial cues that depend on cell-cell interaction and cell-extracellular matrix interaction. Adherens junctions (AJs) and tight junctions (TJs) mediate the cell-cell contact of epithelial cells. Both types form extracellular adhesive contacts between cells and intracellular links to the actin cytoskeleton and signaling pathways, and they do this through different transmembrane proteins [31]. The ILK Signaling (integrin linked kinase) pathway may point to cell-extracellular matrix interaction that takes place during development of cell polarity. Since integrins do not exhibit intrinsic enzymatic activity, binding of integrins to the extracellular matrix proteins, results in recruitment of multiple intracellular proteins that activate signaling cascades and provide links to the actin cytoskeleton, including ILK [32]. ILK has been described to be an important modulator in cell-ECM interactions and the formation of AJs and TJs [33]. Several Rho signaling pathways have been connected to the hESC-RPE (EP and LP) specific dataset. Rho signaling has been implicated in the control of AJ integrity and the maintenance of the AJs [34]. These pathways, together with Actin Cytoskeleton Signaling and Tec Kinase Signaling (involved in actin cytoskeleton signaling), indicate that the hESC-RPE (EP and LP) cells are in the process of cellular remodeling to become a stable layer of epithelial cells.

Bear in mind that these pathways are highly expressed in hESC-RPE (EP and LP) compared to human endogenous RPE. Thus, the hESC-RPE (EP and LP) cells are in the process of epithelial development, while the typical epithelial polarity is already well established in the collected human endogenous RPE.

The most noticeable pathways that are higher expressed in the human endogenous RPE compared to the hESC-RPE are Phototransduction Pathway and The Visual Cycle. *In vivo*, the phototransduction pathway is induced by photon-mediated activation and subsequent destabilization of rhodopsin in the photoreceptors. The adjacent RPE is essential for recycling opsin/all-transretinol back into 11-cis retinal in the coupled (visual) retinol cycle and thus the photoreceptors rely on the RPE for continuing visual phototransduction. It is likely that the *in vivo* laser-dissected RPE samples were contaminated with photoreceptor outer segments, as we observed and discussed extensively elsewhere [3, 19], causing the overexpression of phototransduction genes.

To activate the retinol cycle in the hESC-RPE, physical interaction with the photoreceptor cells is critical. Thus, low expression of these pathways in the hESC-RPE (EP and LP) samples could be caused by the absence of this interactive microenvironment. However, this needs to be tested in future studies.

The human endogenous RPE shows expression of genes within Protein Kinase A (PKA) Signaling, cAMP-mediated Signaling and CREB Signaling in Neurons as shown by IPA. These pathways are intertwined, as CREB is a cellular transcription factor that can be activated by cAMP signaling through PKA. Furthermore, the cAMP-PKA-dependent phosphorylation of CREB affects the expression of Klotho (KL), a gene involved in aging, in RPE physiology and retinal health. KL has important functions in protecting against oxidative stress, in promoting POS phagocytosis by upregulating MERTK gene expression, and in regulating melanogenesis through the genes *MITF* and *TYR* [35]. Interestingly, melatonin levels are reduced in AMD patients and administration of melatonin has been shown to have a protective effect on RPE cells against oxidative stress [36–38]. Accordingly, the gene expression of Melatonin Signaling may also indicate oxidative stress [39]. So, the human endogenous RPE shows increased expression of genes involved in defense mechanisms against oxidative stress as compared to the hESC-RPE cells. This might reflect the age-related enhanced oxidative stress levels *in vivo* [40]*.*

In summary, we show that the *in vitro* hESC-RPE cells are indeed RPE since they show RPE specific morphology and molecular characteristics. We did not find substantial differences in gene expression profiles between EP and LP hESC-RPE, but we did find a clear difference between the hESC-RPE cells and the human endogenous RPE. While they lack the human endogenous RPE expression related to photoreceptor cell presence and defense against oxidative stress, the hESC-RPE cells show expression of pathways that enable the cells to stabilize their epithelial morphology.

## Conclusions

In our study we tried to elucidate to what extent increased pigmentation in hESC-RPE cells relates to differentiation and maturation towards human endogenous RPE. Our data suggest that even though pigmentation seems to be a good biomarker for RPE development, the level of pigmentation does not reflect the maturation state of hESC-RPE. In addition, the data suggest that the hESC-RPE and the human endogenous RPE are substantially different.

Future studies should show whether hESC-RPE cells adopt these functions after transplantation or after growing them on a supporting scaffold that mimics the Bruch’s membrane. Importantly, hESC-RPE cells at early pigmentation stages already show an expression profile representative of differentiated RPE. This suggests that hESC-RPE differentiation procedures for RPE replacement therapies can be shortened significantly which has important implications for the development of new therapeutic strategies in AMD.

## Electronic Supplementary Material

Table S1 (XLS 37 kb)

Table S2 (XLS 51 kb)

Table S3 (XLS 131 kb)

Figure S1 The immunoreactivity of the antibodies was confirmed by immunostaining on human retinal cryosections (RLBP1) and ARPE19 cells on Matrigel coated inserts (MITF, RLBP1, BEST1, ZO1). (TIFF 27050 kb)

High resolution image (GIF 397 kb)

Figure S2 Expression of well-known RPE markers in EP and LP samples by RT-PCR. We show the expression of RAX, VSX2, MITF, TYR, TRPM3, TJP1, RLBP1, RPE65, MERTK and ACTB in four EP samples and four LP samples. (TIFF 267 kb)

High resolution image (GIF 175 kb)

Figure S3 Confirmation of microarray results by sqRT-PCR. We used *ACTB* as the housekeeping gene to normalize the gene expression of the EP and LP samples. We depict the mean and standard deviation for the EP samples in light blue, LP samples in dark blue. We selected genes that were highly expressed in both groups (*RPLP0, LHX2, BASP1, SPOCK1, SLC39A13, SERPINF1, CXCL14, CA14,OTX2*), highly in the EP (*CHD7, FBN2, NCOR1, NDNL2, REST, TMEFF2*) and highly in the LP (*ABHD1, TGFB1*). We found most genes to be in agreement with the microarray results, only *CXCL14, REST, ABHD1, TGFB1* do not show a difference in expression as expected. (TIFF 643 kb)

High resolution image (GIF 40 kb)

Figure S4 sqRT-PCR data of the hESC-RPE cells for the time points 1–8, as defined in Fig. 1. We used *ACTB* as the housekeeping gene to normalize the gene expression in 50 independent differentiation experiments. We depict the mean and the standard deviation for well-known genes involved in the development of RPE cells, *PAX6, OTX2, RAX, VSX2, MITF, BEST1, TRPM3, TJP1, RLBP1, MERTK*. (TIFF 896 kb)

High resolution image (GIF 57 kb)

Figure S5 FACS experiment for phagocytosis of the photoreceptor outer segment by hESC-RPE cells. Twenty-five fresh bovine eyes were obtained from Weza Vlees (Amsterdam, NL) and dissected to obtain the retinas, which were frozen down for storage. Photoreceptor outer segments (POS) were isolated as reported previously using a sucrose gradient [20, 21]. FITC was added to half of the POS (10 mg/mL; A9771, Sigma). Confluent hESC-RPE cell layers on inserts were fed with either medium, medium + POS or medium + POS-FITC (107/cm2) and incubated at 37 °C and 5% CO2 for 16 h. The upper chamber was washed three times with PBS, the cells were trypsinized (0.25% Trypsin-EDTA) for 10 min, washed twice and fixed in 4% PFA for 20 min (in the dark). Cells were washed again, stored in FACS buffer (0.05% NaN3, 5 mg/mL BSA) and level of fluorescence was measured using flow cytometry (BD FACS CantoII, BD Biosciences). All conditions were performed at least in duplo. Several gates were set (Kaluza software) to exclude of dead cells and duplicates. (A) Schematic overview of the experimental setup of the POS phagocytosis experiment. The two scatterplots depict experimental results for the two conditions hESC-RPE + POS and hESC-RPE + POS-FITC. (B) The graph shows the cell count for FITC labeled cells in the three different experimental conditions (in duplo). (C) The flow cytometry gates that were set for this experiment: “Gate A” selects living cells. “Gate B and Gate C” select the single cells and exclude the clumps of cells. “Gate D” selects the fluorescent population. This is the population that is used to plot the graph in B. (TIFF 23057 kb)

High resolution image (GIF 646 kb)
