# Supplementary figures and images for: Stem Cell Derived Retinal Pigment Epithelium: The Role of Pigmentation as Maturation Marker and Gene Expression Profile Comparison with Human Endogenous Retinal Pigment Epithelium
Source: Stem Cell Rev. 2017 Jul 21;13(5):659–69. doi: 10.1007/s12015-017-9754-0 (PMC5602068; doi:10.1007/s12015-017-9754-0)

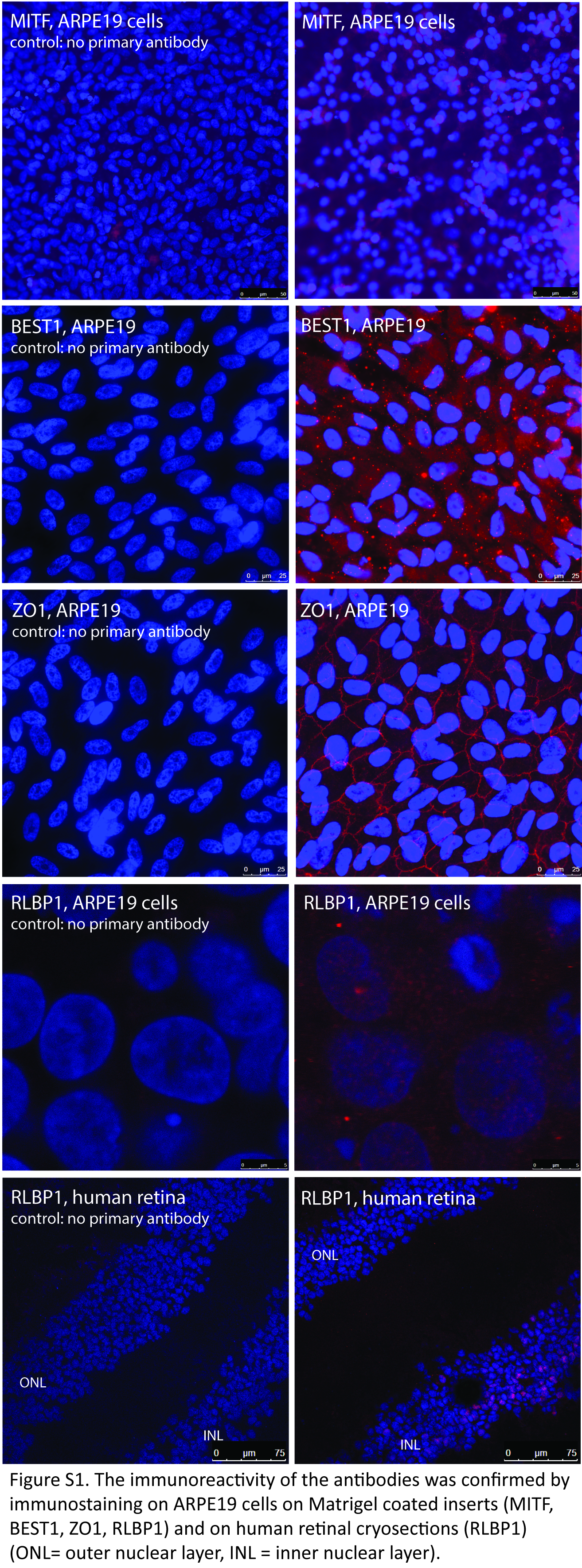

Supplement: Supplementary file 4 — The immunoreactivity of the antibodies was confirmed by immunostaining on human retinal cryosections (RLBP1) and ARPE19 cells on Matrigel coated inserts (MITF, RLBP1, BEST1, ZO1). (TIFF 27050 kb) [file 12015_2017_9754_MOESM4_ESM.tif]

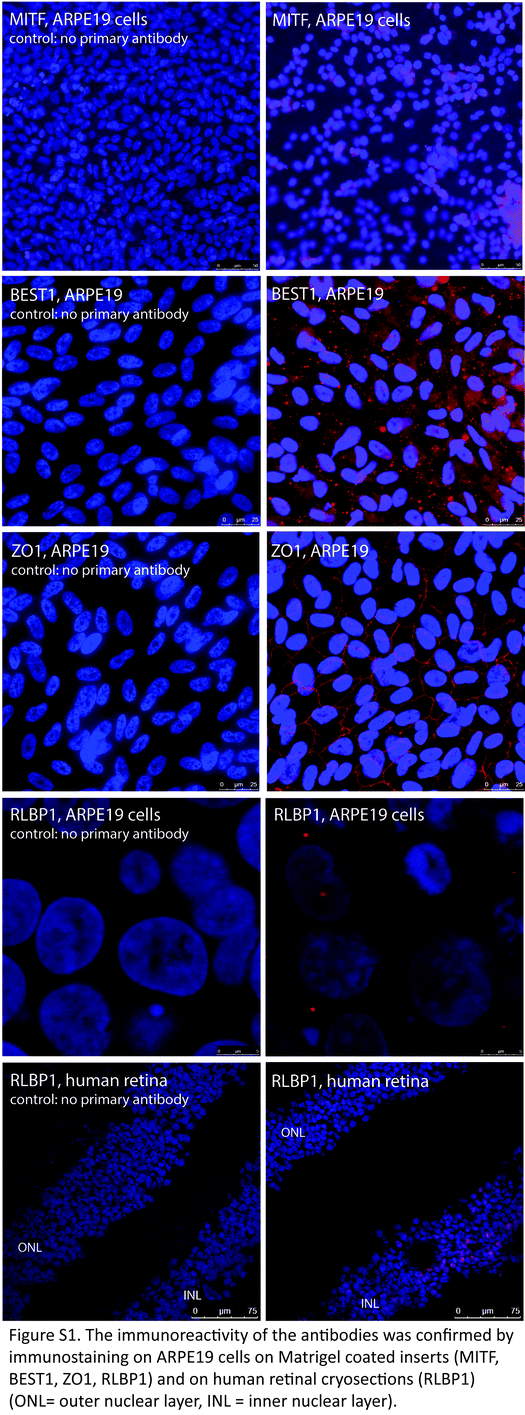

Supplement: Supplementary file 5 — High resolution image (GIF 397 kb) [file 12015_2017_9754_Fig7_ESM.gif]

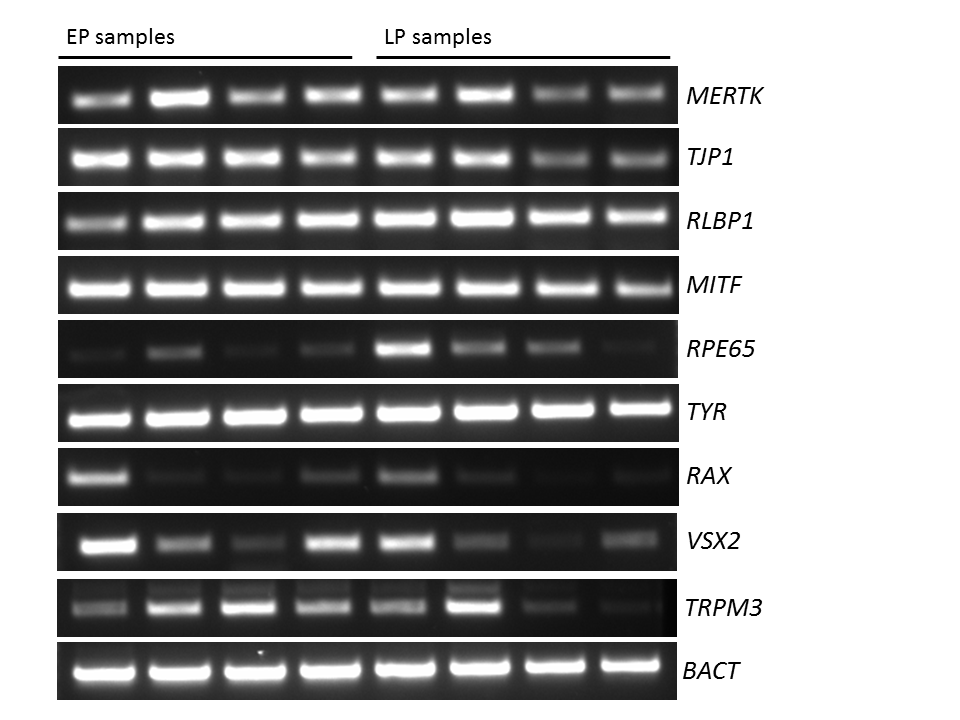

Supplement: Supplementary file 6 — Expression of well-known RPE markers in EP and LP samples by RT-PCR. We show the expression of RAX, VSX2, MITF, TYR, TRPM3, TJP1, RLBP1, RPE65, MERTK and ACTB in four EP samples and four LP samples. (TIFF 267 kb) [file 12015_2017_9754_MOESM5_ESM.tif]

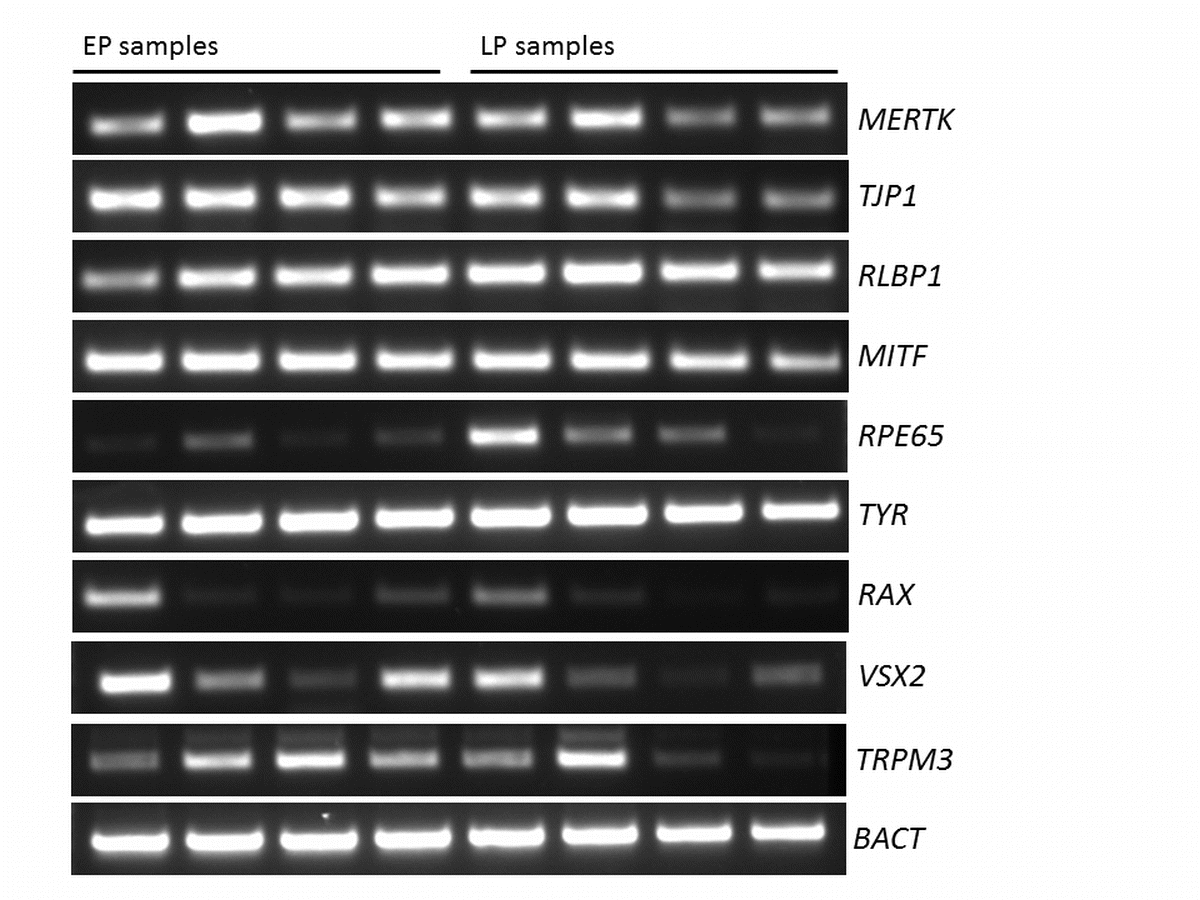

Supplement: Supplementary file 7 — High resolution image (GIF 175 kb) [file 12015_2017_9754_Fig8_ESM.gif]

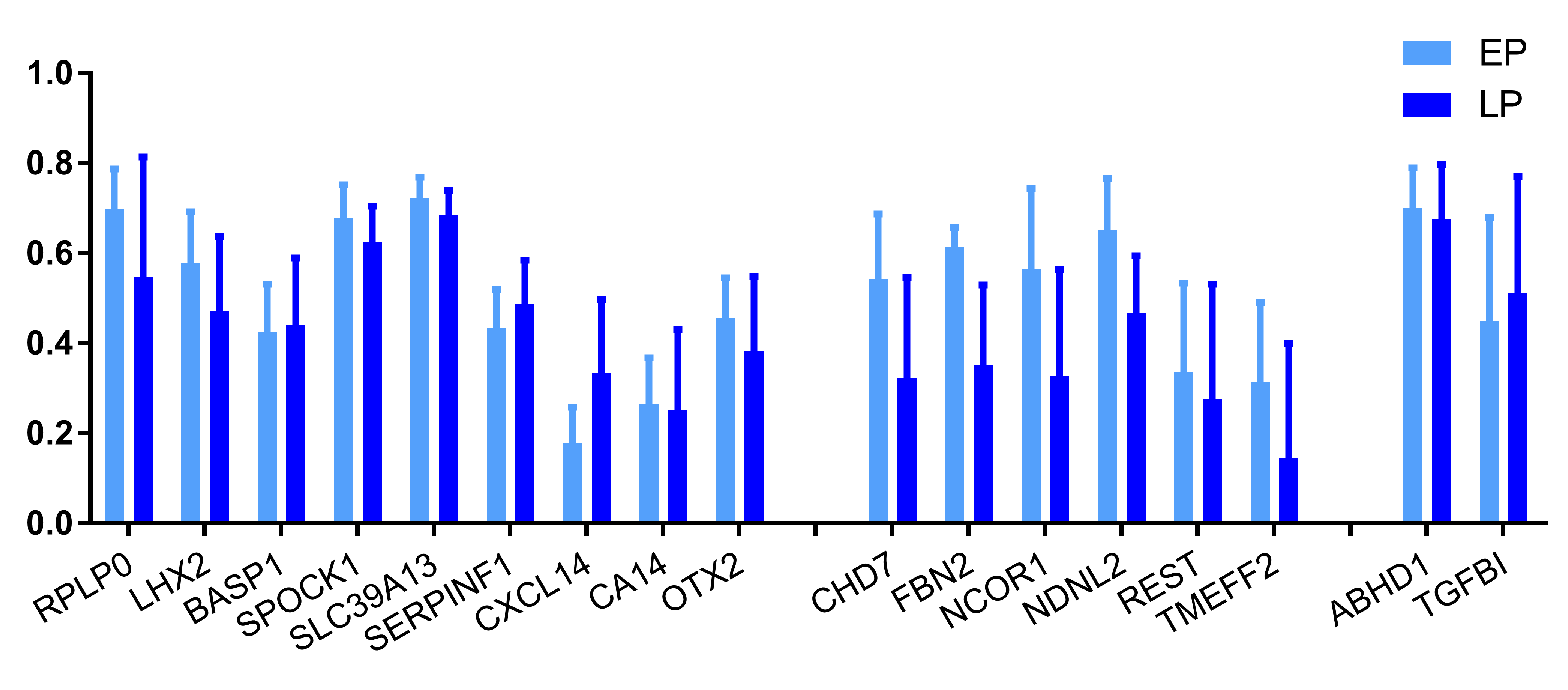

Supplement: Supplementary file 8 — Confirmation of microarray results by sqRT-PCR. We used ACTB as the housekeeping gene to normalize the gene expression of the EP and LP samples. We depict the mean and standard deviation for the EP samples in light blue, LP samples in dark blue. We selected genes that were highly expressed in both groups (RPLP0, LHX2, BASP1, SPOCK1, SLC39A13, SERPINF1, CXCL14, CA14,OTX2), highly in the EP (CHD7, FBN2, NCOR1, NDNL2, REST, TMEFF2) and highly in the LP (ABHD1, TGFB1). We found most genes to be in agreement with the microarray results, only CXCL14, REST, ABHD1, TGFB1 do not show a difference in expression as expected. (TIFF 643 kb) [file 12015_2017_9754_MOESM6_ESM.tif]

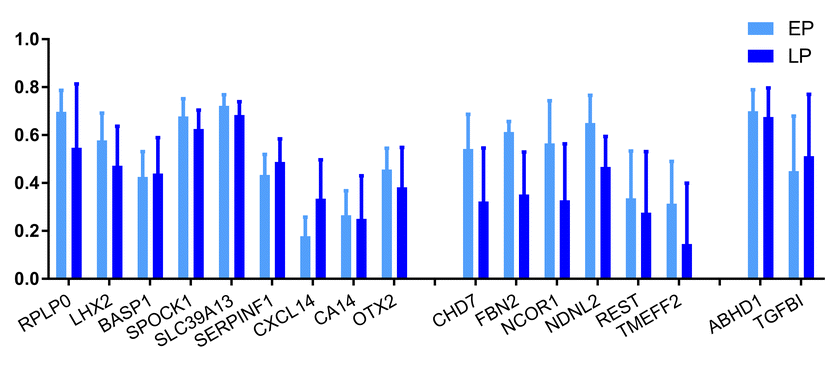

Supplement: Supplementary file 9 — High resolution image (GIF 40 kb) [file 12015_2017_9754_Fig9_ESM.gif]

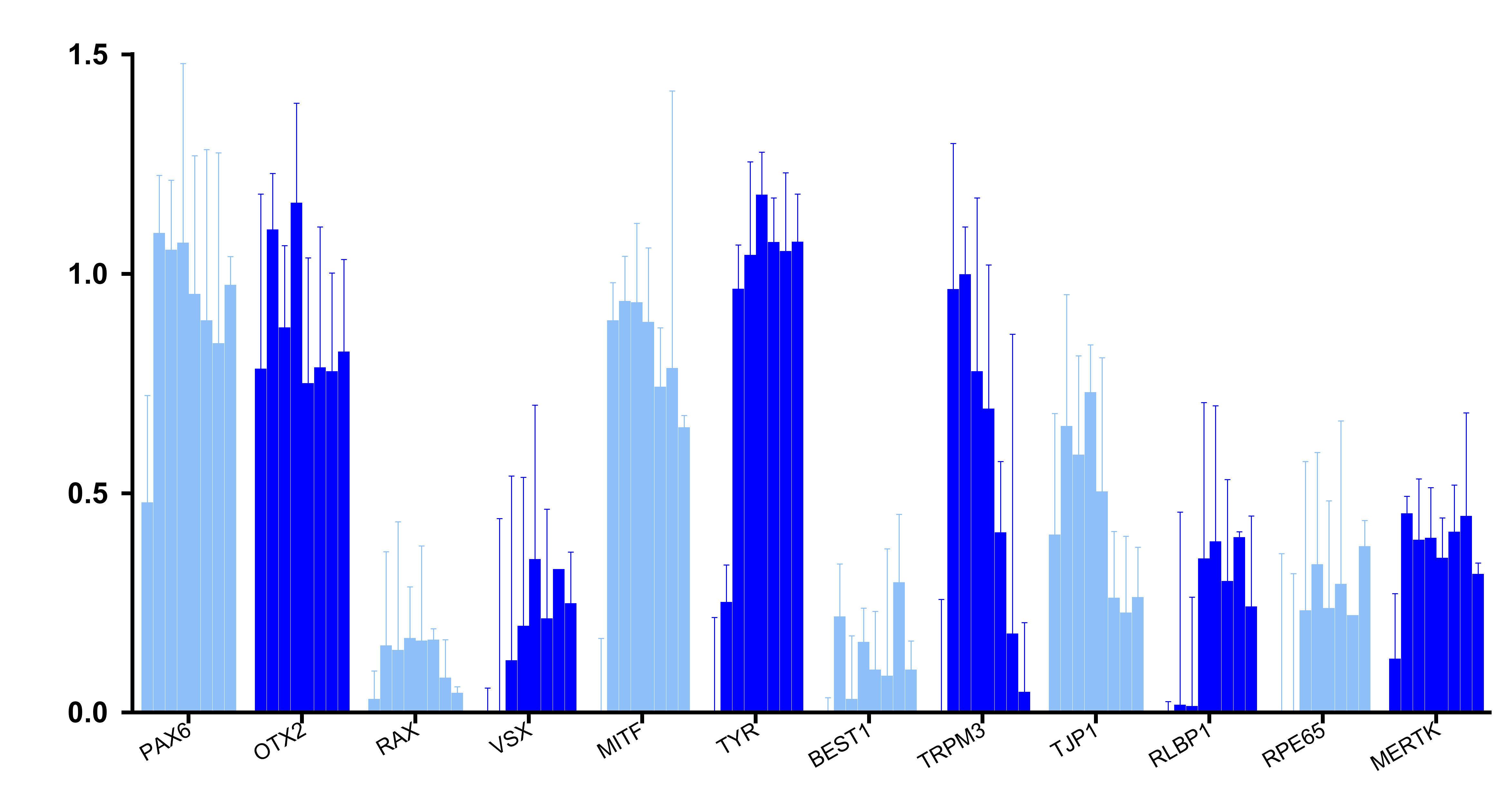

Supplement: Supplementary file 10 — sqRT-PCR data of the hESC-RPE cells for the time points 1–8, as defined in Fig. 1. We used ACTB as the housekeeping gene to normalize the gene expression in 50 independent differentiation experiments. We depict the mean and the standard deviation for well-known genes involved in the development of RPE cells, PAX6, OTX2, RAX, VSX2, MITF, BEST1, TRPM3, TJP1, RLBP1, MERTK. (TIFF 896 kb) [file 12015_2017_9754_MOESM7_ESM.tif]

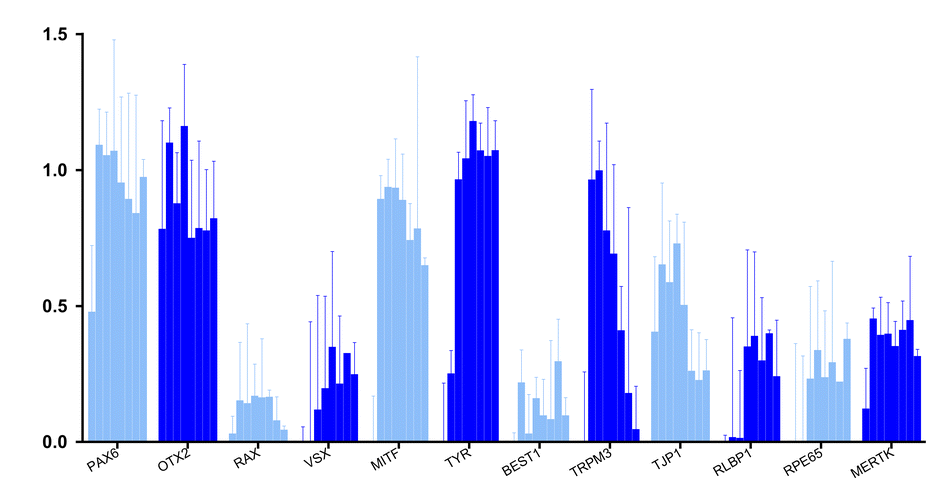

Supplement: Supplementary file 11 — High resolution image (GIF 57 kb) [file 12015_2017_9754_Fig10_ESM.gif]

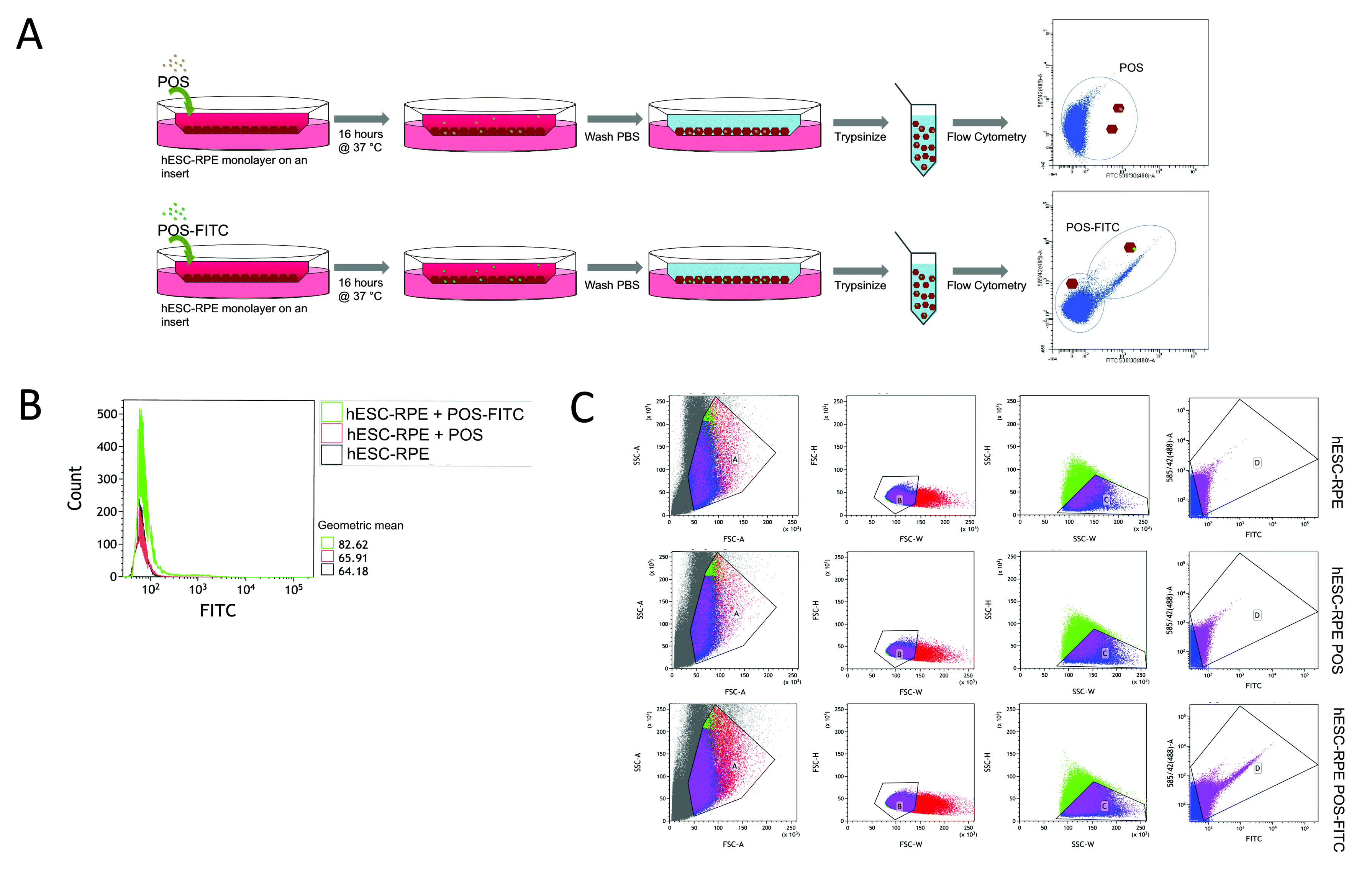

Supplement: Supplementary file 13 — High resolution image (GIF 646 kb) [file 12015_2017_9754_Fig11_ESM.gif]
